# How the brain represents mass

**DOI:** 10.7554/eLife.54373

**Published:** 2020-02-07

**Authors:** Grant Fairchild, Jacqueline C Snow

**Affiliations:** Department of PsychologyUniversity of Nevada RenoRenoUnited States

**Keywords:** physical inference, fMRI, mass, dorsal cortex, machine learning, Human

## Abstract

New fMRI experiments and machine learning are helping to identify how the mass of objects is processed in the brain.

**Related research article** Schwettmann S, Tenenbaum JB, Kanwisher N. 2019. Invariant representations of mass in the human brain. *eLife* **8**:e46619. doi: 10.7554/eLife.46619

Imagine that you are driving to work along an icy road when a deer suddenly jumps into your path. Depending on the distance, you may have time to apply the brakes, or you may consider swerving to avoid a collision. Your intuitive ability to reason about the physics of objects in your environment, for instance their mass, could mean the difference between a fatal crash and a safe arrival at your workplace. However, the way that the brain computes the mass of an object remains a matter of debate. Specifically, we do not know if object mass is primarily processed in dorsal fronto-parietal areas of the cortex (a region involved in action planning), or if this information is first represented in ventral areas of the cortex (which are engaged in object perception).

In 2014 it was reported that activation patterns in ventral visual areas predicted the weight of an object about to be lifted (Gallivan et al., 2014). Conversely, in 2018 one of the present authors (JCS) and co-workers found that a patient with bilateral brain lesions that included the ventral visual cortex was, nevertheless, sensitive to object weight (Buckingham et al., 2018). Now, in eLife, Sarah Schwettmann, Joshua Tenenbaum and Nancy Kanwisher from the Massachusetts Institute of Technology report having characterized the human brain regions and computations involved in intuitive physical reasoning about mass (Schwettmann et al., 2019).

Schwettmann et al. focused on the areas of the fronto-parietal cortex that were identified in a previous study (Fischer et al., 2016). They applied machine learning to fMRI data to characterize how the mass of objects is represented in these brain areas. If an algorithm can be trained to correctly predict whether someone is looking at a heavy or a light object simply based on the patterns of activation in a specific brain region, then it indicates that this brain area actively represents mass. Furthermore, if the algorithm can predict the weight of the object the observer is viewing even when other elements in the stimulus are changed, such as composition or speed, then the representation is said to remain ‘invariant’, or stable. And indeed, Schwettmann et al. show that such invariant representations of object mass exist in the dorsal fronto-parietal cortex across three experiments (Figure 1).

**Figure 1.**
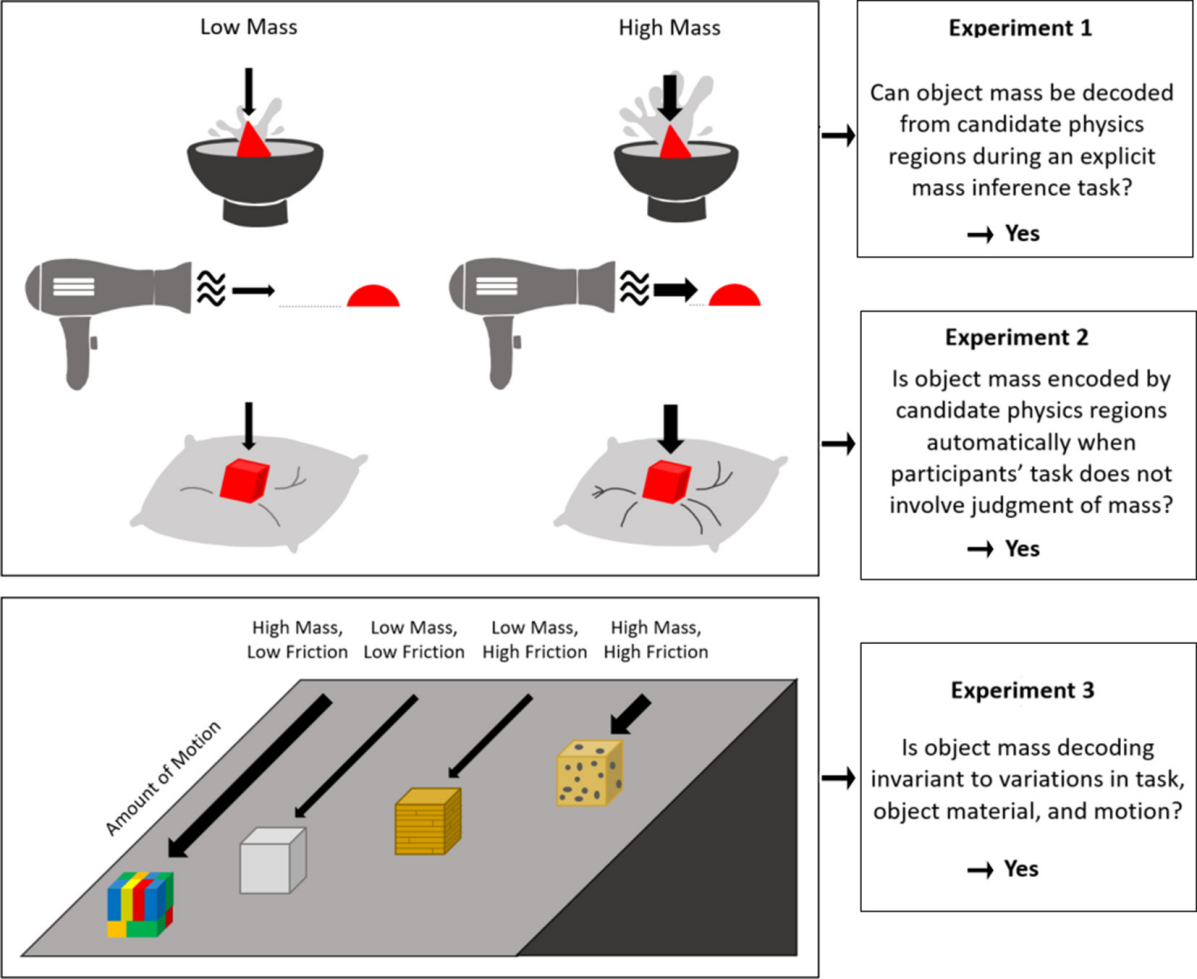
Investigating dorsal representation of object mass. Schematics showing the three experiments performed by Schwettmann et al: in the first experiment, participants watched brief movies depicting basic geometric shapes of low or high mass (left, top). Participants were asked to judge the mass of the object shown in each movie. The second experiment used the same set of movies, except that the participants were required to judge the color of the object in half of the trials. In the final experiment, the geometric solids depicted in the movies were comprised of four different surface materials (lego, aluminum, cardboard, cork) that moved differently when the object slid down a ramp because of differences in mass and friction (left, bottom). Together, the experiments identified dorsal regions that consistently represent object mass, and showed that these representations are both automatic and invariant.

In the first experiment, the participants were asked to judge the weight of basic geometric solids presented in dynamic movie clips in which the objects splashed into water, fell onto a pillow, and were blown across a surface. The algorithm was ‘trained’ on the data obtained from two of these movies — that is, it received both the fMRI data and the information about whether the viewer was observing a heavy or light object. The team then found that the algorithm could predict the weight of the object the volunteer observed in the third movie based solely on the fMRI data from the dorsal brain areas. The second experiment showed that these brain regions also appeared to process mass when the observers were asked to pay attention to the color of the objects rather than their weight. In the last experiment, Schwettmann et al. demonstrated that representations of mass in the dorsal cortex remained invariant even as the surface materials and the amount of motion of the objects changed. Finally, follow-up analyses revealed that the algorithm could reliably use data from the dorsal cortex to predict object mass, but could not do so for data from areas along the ventral cortex.

Taken together, these results reveal that some areas in the fronto-parietal cortex compute physical variables and anticipate the dynamics of objects. The finding that during a perceptual task, object mass is represented in the dorsal cortex but not the ventral areas suggests that information about weight may be processed originally in the dorsal cortex, even though the ventral regions may then receive these signals during action planning.

The results also fit with a growing body of evidence that the dorsal cortex is involved in visual perception as well as space and action computations (Erlikhman et al., 2018; Freud et al., 2016). Exactly how invariant representations of physical parameters, such as object mass, are integrated with the computations required for goal-directed actions remains a tantalizing next step for future research.

Mass representations in the fronto-parietal cortex remain surprisingly invariant across changes in stimuli, environments and tasks. Such invariance is presumably advantageous because mass can be extracted from different visual cues and generalized to new scenarios. That the dorsal cortex computes mass automatically, whether or not it is the focus of someone’s attention, suggests that information about the physical parameters of the environment is sufficiently important for the brain to keep track of it all the time. Future studies will be required to examine whether dorsal brain areas also represent other potentially important physical variables, such as force. It is likely that active, invariant representations of environmental physics can help to quickly guide action, and that they may therefore be a key adaptation for survival.
